# Virulence of Group A Streptococci Is Enhanced by Human Complement Inhibitors

**DOI:** 10.1371/journal.ppat.1005043

**Published:** 2015-07-22

**Authors:** David Ermert, Jutamas Shaughnessy, Thorsten Joeris, Jakub Kaplan, Catherine J. Pang, Evelyn A. Kurt-Jones, Peter A. Rice, Sanjay Ram, Anna M. Blom

**Affiliations:** 1 Division of Medical Protein Chemistry, Department of Translational Medicine, Lund University, Malmö, Sweden; 2 Division of Infectious Diseases and Immunology, Department of Medicine, University of Massachusetts Medical School, Worcester, Massachusetts, United States of America; 3 Section of Immunology and Vaccinology, National Veterinary Institute, Technical University of Denmark, Frederiksberg C, Denmark; 4 Department of Experimental Medical Science, Immunology section, Lund University, Lund, Sweden; Boston Children's Hospital, UNITED STATES

## Abstract

*Streptococcus pyogenes*, also known as Group A Streptococcus (GAS), is an important human bacterial pathogen that can cause invasive infections. Once it colonizes its exclusively human host, GAS needs to surmount numerous innate immune defense mechanisms, including opsonization by complement and consequent phagocytosis. Several strains of GAS bind to human-specific complement inhibitors, C4b-binding protein (C4BP) and/or Factor H (FH), to curtail complement C3 (a critical opsonin) deposition. This results in diminished activation of phagocytes and clearance of GAS that may lead to the host being unable to limit the infection. Herein we describe the course of GAS infection in three human complement inhibitor transgenic (tg) mouse models that examined each inhibitor (human C4BP or FH) alone, or the two inhibitors together (C4BPxFH or ‘double’ tg). GAS infection with strains that bound C4BP and FH resulted in enhanced mortality in each of the three transgenic mouse models compared to infection in wild type mice. In addition, GAS manifested increased virulence in C4BPxFH mice: higher organism burdens and greater elevations of pro-inflammatory cytokines and they died earlier than single transgenic or wt controls. The effects of hu-C4BP and hu-FH were specific for GAS strains that bound these inhibitors because strains that did not bind the inhibitors showed reduced virulence in the ‘double’ tg mice compared to strains that did bind; mortality was also similar in wild-type and C4BPxFH mice infected by non-binding GAS. Our findings emphasize the importance of binding of complement inhibitors to GAS that results in impaired opsonization and phagocytic killing, which translates to enhanced virulence in a humanized whole animal model. This novel hu-C4BPxFH tg model may prove invaluable in studies of GAS pathogenesis and for developing vaccines and therapeutics that rely on human complement activation for efficacy.

## Author Summary

*Streptococcus pyogenes* is an important cause of human infections worldwide, ranging from mild and superficial disease to life-threatening invasive infections. Development of new and efficient therapies for infections requires animal models that faithfully recapitulate infection in humans. Humans are the only natural host of *S*. *pyogenes*; thus, infection in wild-type mice may not reflect infection in humans. Mice that are humanized in ways that are relevant to the studied pathogen would better reproduce human infection. Because *S*. *pyogenes* bind only human, but not mouse complement inhibitors, we used novel strains of humanized mice that produce two human complement inhibitory proteins which allowed us to analyze the impact of human-specific human complement inhibition on the severity of *S*. *pyogenes* infections in mice. Here, we show that expression of human complement inhibitors significantly worsens the outcome of infection in humanized mice. This animal model will permit studies of infection and disease and aid the development of novel therapies and vaccines against *S*. *pyogenes* infections, with emphasis on the human complement system.

## Introduction

*Streptococcus pyogenes*, also known as Group A Streptococcus (GAS) is an important human bacterial pathogen that is widespread and responsible for more than 700 million infections globally each year [1]. GAS causes a spectrum of diseases, ranging from milder pharyngitis and superficial skin infections to more severe illnesses that include acute rheumatic fever (that may be complicated by rheumatic heart disease), post-streptococcal glomerulonephritis and invasive infections. The latter may be accompanied by life-threatening sepsis, streptococcal toxic shock syndrome and/or necrotizing fasciitis [2, 3]. The burden, worldwide, of invasive GAS infection is high, with at least 663,000 new cases and 163,000 deaths each year (25% mortality).

In the absence of effective vaccines against GAS, the outcome of streptococcal infection is determined by the status of the host’s immune system [4]. A key first line of defense against bacterial pathogens involves the complement system, which comprises over 30 soluble proteins and several membrane-associated complement receptors and inhibitors. Complement can be activated on ‘non-self’ cells, such as bacteria, by one or more of three different activation pathways. The classical pathway is initiated by binding of antibodies to the microbial surface, the lectin pathway is triggered by binding of one or more lectins to specific carbohydrate structures and the alternative pathway is activated by a ‘tickover’ mechanism followed by amplification through a positive feedback loop [5]. All three pathways converge at the level of C3 deposition; formation of C3 convertases generates chemoattractant anaphylatoxins and further amplifies deposition of C3 fragments on microbes, which opsonizes the microbial target for efficient phagocytosis. Formation of the lytic membrane attack complex (MAC) may result in direct lysis of gram-negative bacteria. Gram-positive bacteria such as GAS are resistant to MAC-mediated lysis, but are eliminated by phagocytes following opsonization with C3b and iC3b. The complement cascade is tightly regulated by surface bound and soluble inhibitors (or regulators); C4b-binding protein (C4BP) and Factor H (FH) are two examples of the latter which serve to prevent damage to host tissues.

GAS has evolved several virulence factors, which allow the pathogen to colonize its human host, escape the immune system and successfully establish infection [6, 7]. GAS infection is human-specific; in the context of its interaction with the innate immune system, GAS interacts with several human proteins, including fibrinogen, albumin and the Fc portion of IgG. Fibrinogen binding to GAS reduces opsonization, while IgG Fc binding to GAS may prevent recognition by phagocyte Fc receptors [8, 9]. GAS surface molecules that are important for these interactions include the M protein and other members of the M protein family [10]. M protein family members share high DNA sequence identity (>70%), but are encoded by different genes (*enn*, *mrp*, *fcrA*, *arp*, *protH* and others; reviewed in [11]). Certain M or M-like proteins mediate GAS binding of human C4BP and/or human FH [12, 13]. A particularly virulent GAS strain called AP1 binds human C4BP and FH through protein H, which is a member of M protein family [14–16]. Studies *in vitro* have shown that inhibition of complement activation through surface bound human FH and C4BP enables GAS to evade opsonization [17]. However, *in vivo* evidence implicating C4BP and Factor H in GAS infections has been lacking because a suitable animal model has not been tested.

Several GAS bind only human, but not mouse C4BP and/or FH [18]. Thus, wild-type mouse models are not suitable to evaluate the roles of these human complement inhibitors in GAS infection. To circumvent these limitations *in vivo* [19], we have employed novel transgenic mice that express human C4BP and FH.

## Results

### Generation of mice transgenic for human complement inhibitors

Complement activation plays a key role in clearance of certain GAS by phagocytes [20]. The binding of serum complement inhibitors to bacterial surfaces regulates complement activation. Certain GAS bind human C4BP (hu-C4BP) and human FH (hu-FH) exclusively, but not the corresponding mouse complement inhibitors. Therefore, we hypothesized that mice that express these human complement inhibitors would manifest increased severity of infection with GAS compared to wild type mice.

**Fig 1:**
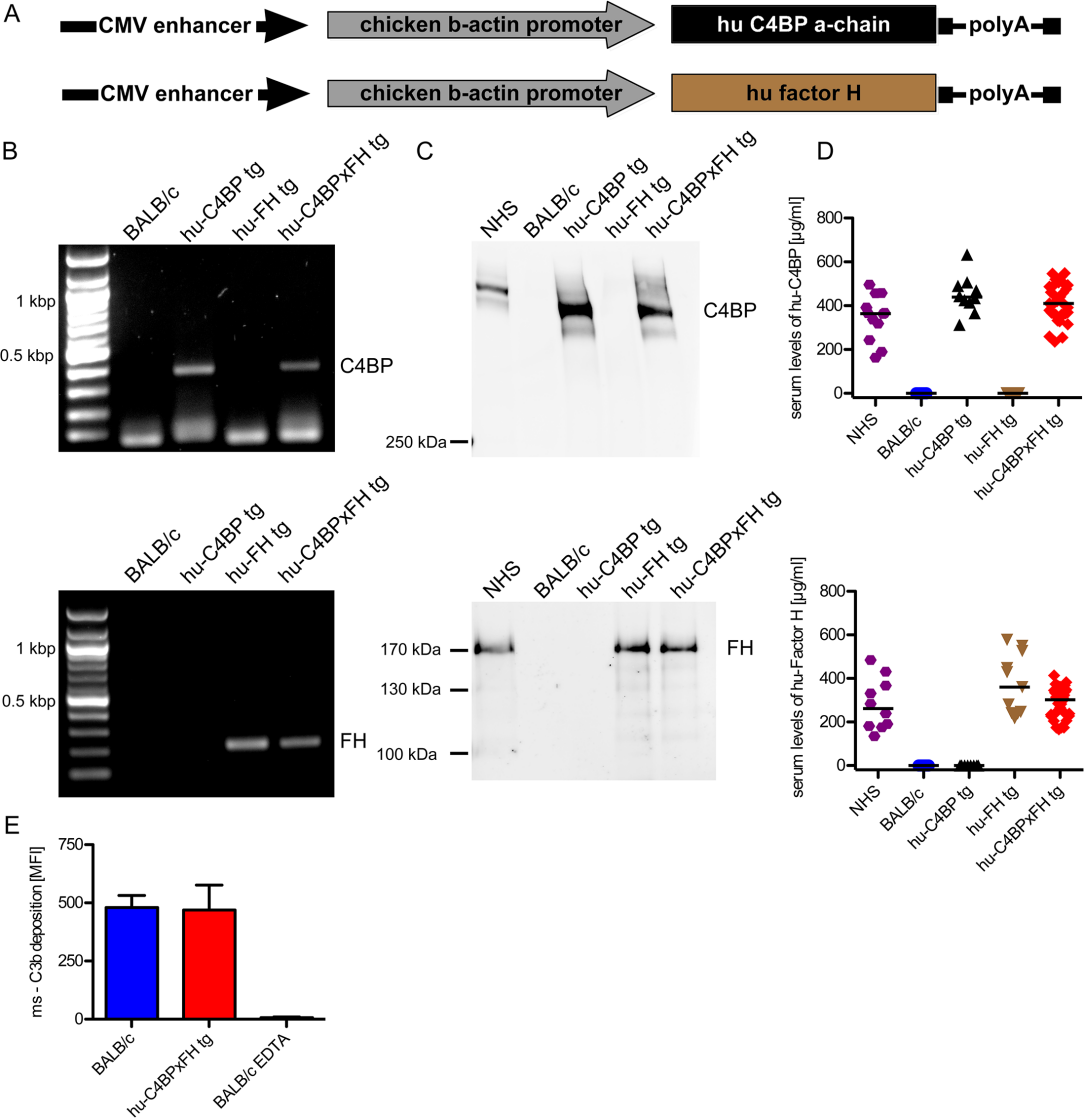
Construction of hu-C4BP, hu-FH and C4BPxFH tg BALB/c mice. (**A**) The depicted constructs were used to generate transgenic animals that expressed human C4BP and/or FH in addition to their endogenous counterpart C4BP and FH molecules. (**B**) PCR analysis confirmed the presence of the either hu-C4BP α-chains (upper panel), human Factor H (FH) (lower panel) or both (both panels) in the tg animals, but not in BALB/c wt mice. **(C)** SDS-PAGE and western blot analysis confirmed that hu-C4BP (**C**, upper panel non-reduced gel) and hu-FH (**C**, lower panel, reduced gel) were detectable in the appropriately designated tg, but not in BALB/c mouse serum. (**D**) Serum levels of hu-C4BP and hu-FH were determined using a sandwich-ELISA. (**E)** BALB/c (blue) and hu-C4BPxFH tg (red) serum (20%) deposit similar amounts of C3 on zymosan particles by flow cytometry. EDTA-treated BALB/c serum (negative control) did not deposit any C3 on zymosan (negative control; n = 3 sera from individual animals). Statistical analysis: Kruskal-Wallis analysis with Dunn’s post-test (**D**) and 1-way ANOVA with Bonferroni’s post-test (**E**).

The α-chain of hu-C4BP was cloned into a pCAGS vector (Fig 1A), which was then used to generate hu-C4BP transgenic animals in a BALB/c background. Using a similar approach, previously we had generated hu-FH tg mice in a BALB/c background, (Fig 1A and [21]). Hu-C4BPxFH tg animals were generated by crossing hu-C4BP and hu-FH single transgenic animals. These mice also express endogenous mouse FH and C4BP. Genotyping confirmed the presence of the human genes in the respective tg animals (Fig 1B; C4BP, upper panel and FH, lower panel). Western blot analysis confirmed expression of the human proteins in the corresponding strains of mice (Fig 1C; C4BP, upper panel and FH, lower panel). As expected, hu-C4BP protein in tg mouse serum displayed a lower molecular mass compared to C4BP in normal human serum (NHS) because these mice lack the human C4BP β-chain gene. The hu-C4BP molecule lacking the β-chain (as expressed by our tg animals) is fully functional as a complement inhibitor (see below; [22]). Human FH expressed by tg mice migrated in a manner similar to FH present in NHS on SDS-PAGE. ELISA measurements of both human inhibitors in mouse serum with antisera specific for human FH and C4BP revealed levels that were comparable to those in NHS (Fig 1D; C4BP, upper panel and FH, lower panel). To ensure that activation of the mouse complement system in hu-C4BPxFH tg serum was relatively unimpaired on a complement activator surface, we compared mouse C3 deposition on zymosan particles (zymosan is an activator of the alternative pathway of complement [23]) using BALB/c and hu-C4BPxFH tg serum. Both sera at concentrations of 20% deposited similar amounts of mouse C3 on zymosan, indicating that the complement system in ‘double’ transgenic mouse serum was not unduly inhibited by concomitantly expressed human complement inhibitors (Fig 1E). Experiments using 50% and 100% serum concentrations also did not show any differences between wt and tg sera.

To exclude major defects in the major innate immune pathways in the tg animals, we compared the ability of wt and C4BPxFH tg macrophages to respond to infection by culturing peritoneal macrophages with several different TLR and cGAS stimulating ligands including LPS (TLR4 ligand), Pam2CSK4 (TLR2 ligand), cytosolic dsDNA (lipofectamine + dAdT, STING ligand), Sendai virus (RIG-I ligand), live Gram-positive (GAS AP1) and Gram-negative bacteria (*Neisseria gonorrhoeae*; N.G.). We collected supernatants after 18h and measured IL-6 secretion to assess NF-κB activation and RANTES (an IFN-stimulated gene) secretion to assess TRIF/STING activation. Levels of IL-6 and RANTES were similar in all tested animals (S1 Fig) confirming that expression of the human tg proteins did not affect innate immune signaling networks for cytokine synthesis. Taken together, expression of hu-C4BP and hu-FH in tg mice does not result in any apparent immune defects.

### Human C4BP and FH diminish C3 fragment deposition on GAS from mouse serum

**Fig 2:**
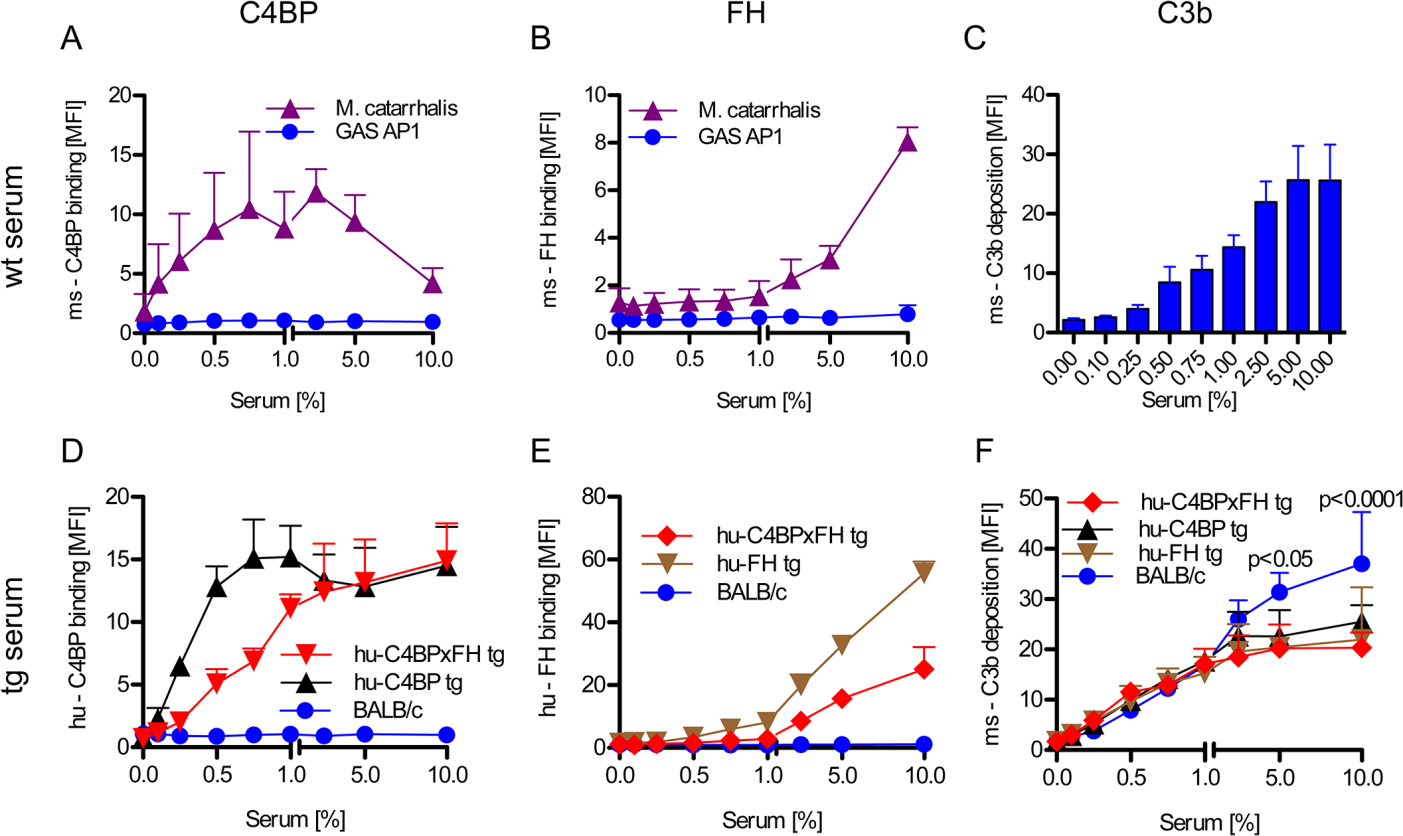
Human complement inhibitors decrease opsonization of GAS. GAS AP1 was incubated either in increasing amounts of BALB/c wild type serum (**A**-**C**), hu-C4BP, hu-FH or hu-C4BPxFH tg serum (**D-F**) prior to FACS analysis of surface bound: ms (mouse) C4BP (**A**); hu-C4BP (**D**); ms-FH (**B**); hu-FH (**E**) or ms-C3b deposited on GAS (**C**, **F**). GAS showed significantly more C3b deposition when incubated in BALB/c wt mouse serum (**C**) vs. incubation in hu-C4BPxFH tg serum (**F**). *M*. *catarrhalis* bound ms-C4BP and ms-FH (**A**,**B**) and served as a positive control. All results are expressed as the Mean ± SD. of three independently performed experiments. Statistical analysis: 2-way ANOVA with Bonferroni’s post-test (**F**).

Evading complement attack through binding of host inhibitors to prevent opsonization can be an early and crucial step in the pathogenesis of GAS (reviewed in [6]). Activation of the complement system marks the pathogen for removal. Certain GAS bind hu-C4BP and hu-FH but not the mouse counterparts (Fig 2A and 2B). Wild-type mouse serum complement is activated on GAS strain AP1 and results in C3 fragment (C3b/iC3b) deposition on the bacterial surface in a dose-dependent manner (Fig 2C).

GAS strain AP1 binds hu-C4BP and hu-FH to its surface via protein H, a member of the M-protein family [12, 15]. Consistent with prior data, bacteria incubated in sera from both hu-C4BP and hu-C4BPxFH tg animals bound hu-C4BP in a dose dependent manner (Fig 2D). Similarly, we detected surface bound hu-FH on bacteria incubated in hu-FH and hu-C4BPxFH tg sera (Fig 2E). As expected, neither hu-C4BP nor hu-FH were detected on GAS incubated in wild type BALB/c serum (Fig 2D and 2E; blue line).

Consistent with the ability of hu-C4BP and hu-FH to inhibit mouse complement, bacteria incubated in hu-C4BP, hu-FH or hu-C4BPxFH tg mouse sera showed significantly reduced C3 fragment deposition compared to wt BALB/c serum at serum concentrations ≥5% (Fig 2F). These results provide evidence *in vitro* of the importance of the binding of soluble human complement inhibitors to limit C3 deposition and opsonization.

### Decreased opsonization diminishes uptake by professional phagocytes

**Fig 3:**
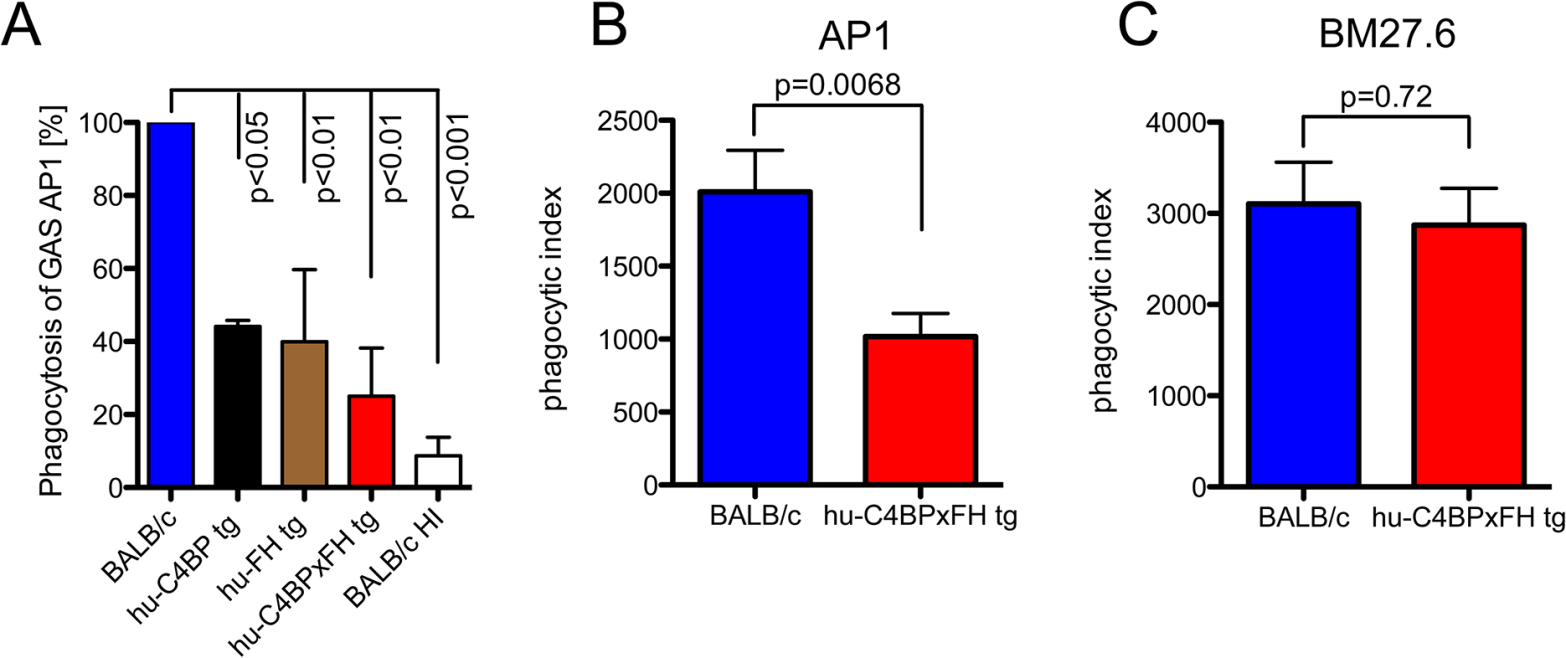
Phagocytosis of GAS AP1 is reduced in the presence of hu-C4BP and hu-FH. (**A**) Binding of hu-FH and hu-C4BP decreases opsonophagocytosis of GAS strain AP1 *in vitro*. Strain AP1 was incubated with bone marrow derived macrophages from wild-type BALB/c mice in the presence of wild type, heat inactivated wild type (BALB/c HI), hu-C4BP, hu-FH or hu-C4BPxFH tg serum. Uptake of AP1 in BALB/c (wild type) serum was set to 100%. (**B**) and (**C**). Binding of hu-FH and hu-C4BP decreases opsonophagocytosis of AP1 *in vivo*. BALB/c and hu-C4BPxFH tg mice were injected i.p. with Carboxyfluorescein succinimidyl ester (CFSE)-labeled GAS strain AP1 (**B**; 5 BALB/c and 8 hu-C4BPxFH tg mice) or its isogenic mutant GAS strain BM27.6 that lacks expression of M protein (and therefore protein H) and neither binds hu-FH or hu-C4BP (**C;** 6 BALB/c and 5 hu-C4BPxFH tg mice). Phagocytic index was calculated as the product of the % of neutrophils in the peritoneal cavity and % of those neutrophils containing CFSE labeled GAS (see also S3 Fig where these measurements are shown separately). Results are expressed as Mean ± SEM of at least three independently performed experiments. Statistical analysis: 1way ANOVA with Dunnetts post-test (**A**) and students t-test (**B**-**C**).

The data above demonstrates that hu-C4BP and hu-FH limit C3 deposition on GAS strain AP1. To assess the impact of these two human complement inhibitors on phagocytosis, we infected mouse bone marrow derived macrophages *in vitro* with GAS strain AP1 in the presence of mouse sera with and without different human complement inhibitors. The presence of hu-C4BP or hu-FH decreased phagocytosis by more than 65%. Both inhibitors together reduced bacterial uptake by 75% compared to wild type mouse serum lacking human complement inhibitors (Fig 3A).

To determine whether the presence of hu-C4BP and hu-FH affected GAS opsonophagocytosis *in vivo*, we infected wt and hu-C4BPxFH tg mice with strain AP1 i.p. and harvested peritoneal cells 2 hours post-infection. Using flow cytometry we identified the proportion of neutrophils in peritoneal exudate cells (S2 Fig shows the gating strategy). We found that in wt BALB/c animals infected with GAS strain AP1, more than 55% of all cells obtained were neutrophils, while significantly fewer neutrophils were recruited in hu-C4BPxFH tg animals during infection (S3A Fig). As a control we infected wt and hu-C4BPxFH tg mice infected with GAS mutant strain BM27.6 that is unable to bind either hu-C4BP or hu-FH (Table 1). Strain BM27.6 recruited similar amounts of neutrophils in both types of animals (S3B Fig). Notably, AP1 uptake by neutrophils from BALB/c mice was significantly higher than that seen in hu-C4BPxFH tg mice (S3C Fig) while BM27.6 uptake by neutrophils was similar in BALB/c and hu-C4BPxFH tg mice (S3D Fig). We calculated a phagocytic index, which multiplies the proportion of neutrophils recruited to the peritoneum times the percent of neutrophils that ingest bacteria. The phagocytic index of AP1 infected BALB/c wild type mice was 2-fold higher than the index in hu-C4BPxFH tg animals, indicating that binding of the complement inhibitors influences the uptake of AP1 (Fig 3B). The phagocytic indices of the two mouse strains that were infected with BM27.6 were similar (Fig 3C), consistent with the inability of BM27.6 to bind to hu-FH or hu-C4BP (S3 Fig and [15]). Taken together, hu-C4BP and hu-FH expressed in mouse serum bind to strain AP1; decrease mouse C3 fragment deposition on the bacterial surface, which leads to diminished recruitment of phagocytes and reduced phagocytosis both *in vitro* and *in vivo*.

**Table 1:** GAS strains used and their relevant characteristics.

Strain	Description	Ligand for complement inhibitor [Ref.]		covRS genotype
		Hu-C4BP	Hu-FH	
AP1	Wild type strain, WHO reference strain; expresses M1	protein H [25, 26]	protein H [15, 25]	Mutated covS
BM27.6	Isogenic mutant of AP1, lacks protein H and M protein	*No binding* [15, 26]	*No binding* [15]	Mutated covS
AP3	Wild type strain; expresses M3	*No binding* [27, 28]	*No binding* [28]	No mutation
AP18	Wild type strain; expresses M18	Enn 18 [27, 29]	M18 [28, 29]	No mutation

### Complement inhibition enhances lethality of GAS infection

**Fig 4:**
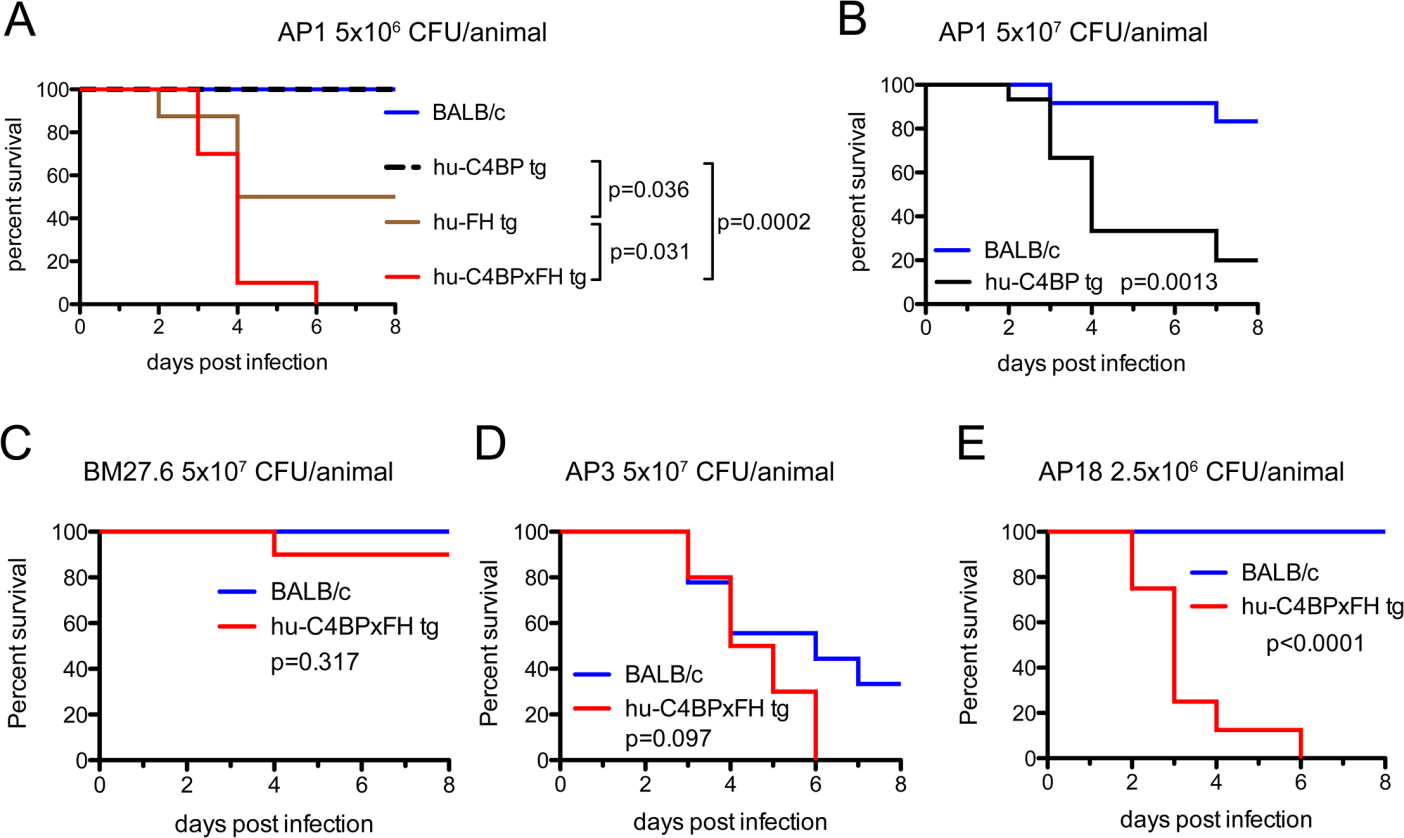
Human complement inhibitors worsen GAS AP1 infection. (**A**) Strain AP1 is more virulent in tg mice that express human complement inhibitors. BALB/c (n = 7), hu-C4BP tg (n = 9), hu-FH tg (n = 8) and hu-C4BPxFH tg (n = 7) mice were infected i.v. with 5x10^6^ CFU of GAS AP1. (**B**) At high-dose infection with strain AP1 GAS (5x10^7^ CFU/mouse i.v.), ~83% of wt mice (n = 12) survived for 8 days compared to 20% survival of C4BP (single) tg mice (n = 15). The presence of hu-FH and hu-C4BP together increased the virulence of strain AP1. (**C**) Isogenic mutant strain BM27.6 (derived from AP1 and lacks protein H and does not bind hu-FH or hu-C4BP) was avirulent (no mortality) in wt BALB/c with a single fatality in hu-C4BPxFH tg mice. Wild-type BALB/c (n = 10) and hu-C4BPxFH tg (n = 10) mice were infected i.v. with 5x10^7^ CFU GAS BM27.6. (**D**) hu-C4BPxFH tg mice (n = 9) and wt BALB/c mice (n = 10) infected with 5x10^7^ CFU strain AP3 (expresses M3 protein that does not bind either hu-FH or hu-C4BP) did not differ significantly in their times to death. (**E**) GAS strain AP18 (expresses M18 protein that binds both hu-FH and hu-C4BP) infection showed decreased time to death in hu-C4BPxFH tg mice compared to infection in BALB/c control mice. Eight mice in each group were infected with 2.5x10^6^ CFU of strain AP18. Statistical analyses shown in all graphs was performed using the Mantel-Cox test.

We next asked whether human complement inhibitors affected the survival of mice infected with GAS. We infected single transgenic hu-C4BP, hu-FH mice and double tg hu-C4BPxFH tg mice intravenously (i.v.) and monitored animals for signs of disease for 8 days. Based on *in vitro* data and the results of *in vivo* phagocytosis experiments, we hypothesized that double tg mice would be more susceptible to GAS infection with human complement inhibitor binding GAS strains (hu-FH- and hu-C4BP-binding) than single tg and normal control mice. Indeed, we observed significant differences across single hu-tg and double-C4BPxFH tg mouse strains: C4BPxFH tg mice were the most susceptible to lethal GAS disease caused by hu-inhibitor binding strains. At a dose of 5x10^6^ CFU/mouse (i.v.), both single C4BP tg and wt animals survived for 8 days and showed no signs of disease (Fig 4A, blue and dotted black line, respectively); hu-FH tg animals were more susceptible than wt or C4BP tg mice with a median survival of 6.5 days and a 50% fatality rate at 8 days (Fig 4A, brown line). At high-dose infection with strain AP1 GAS (5x10^7^ CFU/mouse i.v.), ~83% of wt mice survived for 8 days compared to 20% survival of C4BP (single) tg mice (Fig 4B). Hu-C4BP tg mice showed a median survival of only 4 days. Notably, BALB/c mice are relatively resistant to infections with GAS, necessitating high inocula to induce disease in wt [24] and single C4BP tg mice. Transgenic animals that expressed both hu-FH and hu-C4BP were the most susceptible and all mice given the lower dose (5x10^6^ CFU/mouse i.v), died within 6 days of inoculation (Fig 4A, red line). These data indicate that simultaneous inhibition of the classical and alternative pathways on the bacterial surface by hu-C4BP and hu-FH, respectively, greatly enhances GAS strain AP1 virulence and highlights the importance of regulation of complement activation by the bacteria. Because hu-C4BP and hu-FH together displayed an additive effect in down-regulating complement in mice and were the most susceptible to lethal infection, we performed all subsequent experiments using hu-C4BPxFH tg mice.

We hypothesized that the increased lethality observed in the experiments above would not be unique to GAS strain AP1 (binds hu-C4BP and hu-FH through protein H) and tested additional GAS strains in our animal model (listed in Table 1). We also examined whether the mortality-enhancing effects of the two human complement inhibitors were restricted only to GAS strains that bound hu-C4BP and hu-FH and determined the ability of these bacterial strains to survive infection.

We first infected BALB/c and hu-C4BPxFH tg animals with GAS strain BM27.6, an isogenic mutant of AP1, lacking both M protein and protein H, or with the wild-type strain AP3 strain (Table 1). Neither BM27.6 nor AP3 bind hu-C4BP or hu-FH (S4 Fig). All 10 BALB/c and 9 out of 10 hu-C4BPxFH tg mice infected with 5x10^7^ CFU BM27.6 survived (Fig 4C). Infections with either 1x10^7^ or 5x10^8^ CFU BM27.6 also revealed no difference in mortality between 10 BALB/c and 10 hu-C4BPxFH tg mice (S5A and S5B Fig). Although infections with GAS AP3 at an inoculum of 5x10^7^ CFU/animal produced disease in both wt and hu-C4BPxFH tg mice, differences in survival across groups was not significant (67% mortality at day 8 in BALB/c and 100% in hu-C4BPxFH tg; Fig 4D). Lower (2x10^7^) and higher (1x10^8^) inocula of AP3 also showed similar mortality in both groups (S5C and S5D Fig). By contrast, GAS AP18, which like AP1, binds both hu-C4BP and hu-FH, showed significantly increased virulence in hu-C4BPxFH tg compared to wt mice; like AP1, all AP18-infected animals had died by 6 days, while all wt BALB/c control mice survived (Fig 4E) and did not show signs of morbidity. Using 4 strains of GAS (2 that bind C4BP and FH and 2 that do not), these results indicate that GAS strains that bind these complement inhibitors show significantly increased virulence in mice that express human transgenes for both of the inhibitors, singly or in combination.

### Human complement inhibitors exacerbate GAS infections in mice

**Fig 5:**
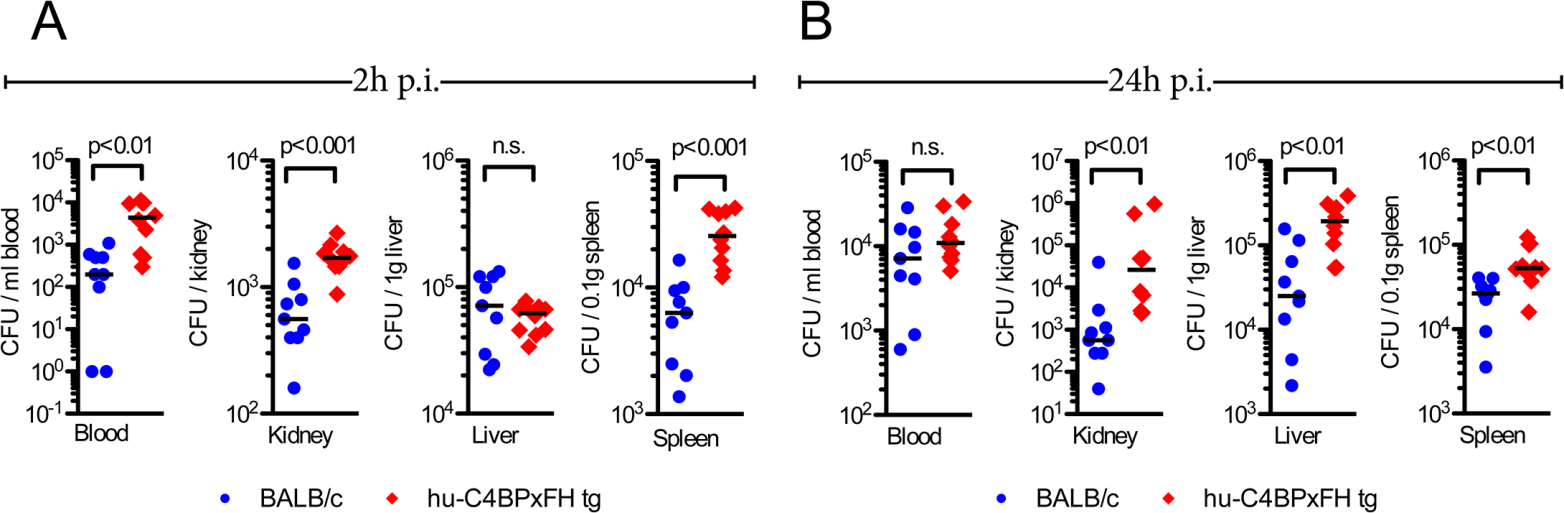
Hu-C4BPxFH tg mice show increased bacterial dissemination in organs. BALB/c wt and hu-C4BPxFH tg mice were infected i.v. with GAS AP1. To assess bacterial dissemination to organs, BALB/c wt and hu-C4BPxFH tg mice were infected i.v. with 5x10^6^ GAS AP1. Nine BALB/c and 10 hu-C4BPxFH tg animals were sacrificed at (**A**) 2h and at (**B**) 24h after infection. CFUs enumerated from the indicated organs are shown. Individual samples from each group and the corresponding medians are shown. The Mann-Whitney test was used to compare groups.

We next quantified the bacterial burden in the blood, kidneys, liver and spleen of hu-C4BPxFH and BALB/c mice infected with AP1 GAS. Mice were sacrificed either at 2h or 24h post-infection and organs were homogenized and plated to enumerate bacterial CFUs. As early as 2h, we noted significantly higher bacterial loads (up to 1.5 log_10_ higher) in blood, kidney and spleen of hu-C4BPxFH (‘double’) tg mice compared to wt BALB/c animals; liver samples from both strains showed similar bacterial loads (CFUs) (Fig 5A). At 24h post-infection, the liver, spleen and kidneys of hu-C4BPxFH tg mice showed significantly greater bacterial loads compared to loads in BALB/c mice (Fig 5B). In contrast to bacterial loads in the organs, bacterial loads from wt BALB/c blood were similar to levels in the blood of hu-C4BPxFH mice (Fig 5B). The greater bacterial burden in hu-C4BPxFH mice early in the course of infection points to altered innate immune defenses, which may have been the result of decreased opsonophagocytotic potential of GAS in tg animals (Fig 3 and S3 Fig). Taken together, GAS avoids early phagocytic clearance and establishes a more severe invasive infection in the transgenic animals.

### Enhanced GAS infection is accompanied by increased cytokine release

**Fig 6:**
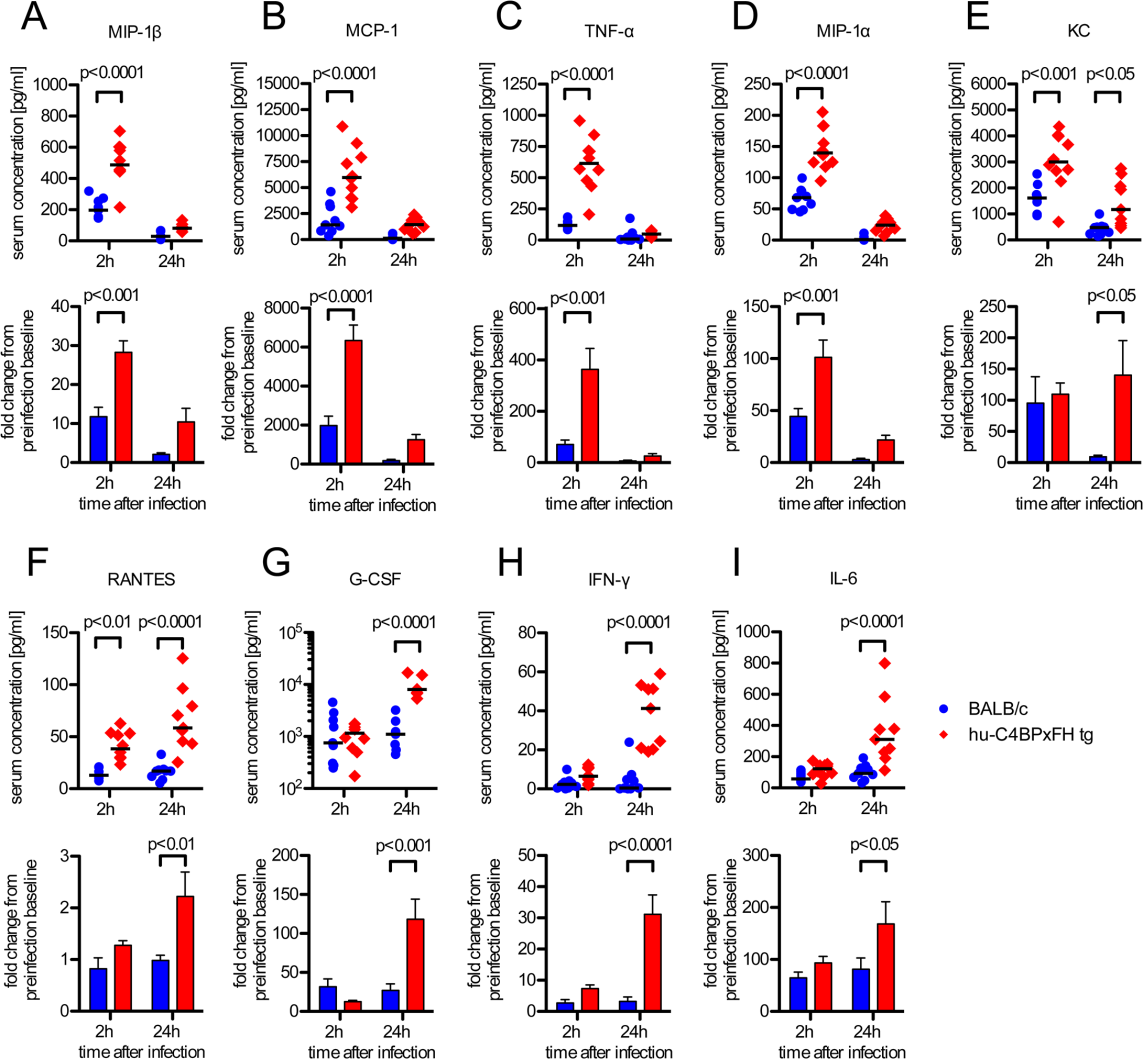
GAS induced sepsis enhances systemic cytokine release in hu-C4BPxFH tg mice. Animals were infected i.v. with 5x10^6^ GAS AP1. (**A**-**I**) Nine BALB/c and 10 hu-C4BPxFH tg animals were sacrificed at 2h, and the remaining animals were sacrificed at 24h after infection and sera obtained for cytokine analysis. Cytokine levels of individual samples (with medians indicated) are shown (1^st^ and 3^rd^ rows) as well as fold change compared to baseline levels obtained prior to infection from individual animals (2^nd^ and 4^th^ rows) (bar graphs; means ±SEM). Statistical analysis: 2-way ANOVA with Bonferroni’s post-test.

Sepsis typically is associated with highly elevated levels of serum cytokines that lead to the systemic inflammatory response syndrome (SIRS) or cytokine storm, which often precedes multi-organ failure and eventually death [30]. We analyzed serum cytokines during the course of infection. Based on initial screening for 23 different serum cytokines in infected BALB/c and C4BP tg animals, we selected the following 11 cytokines for further analysis: IL-1β, IL-6, IL-13, G-CSF, IFN-γ, KC, MCP-1, MIP-1α, MIP-1β, RANTES and TNF-α. Using a multiplex analysis for these 11 cytokines, we analyzed serum samples from C4BPxFH tg and BALB/c wt mice 24h prior to, as well as 2h and 24h post infection. At 2h after infection we identified significantly increased serum levels of MIP-1β, MCP-1, TNF-α and MIP-1α in hu-C4BPxFH compared to wt mice (Fig 6A–6D). After 24h we observed a shift in the cytokine pattern with MIP-1β, MCP-1, TNF-α, KC and MIP-1α becoming strongly down regulated. In addition to KC and RANTES, which remained significantly higher in transgenic mice both at 2h and 24h post-infection (Fig 6E and 6F), MIP-1β, MCP-1, TNF-α, MIP-1α and KC peaked at 2h post-infection; levels of MIP-1β, MCP-1, TNF-α, MIP-1α and KC were also significantly increased in hu-C4BPxFH tg compared to wt mice (Fig 6A–6E) at 2h post-infection. G-CSF, IFN-γ and IL-6, exhibited similar levels in BALB/c and C4BPxFH tg mice at 2h but were elevated significantly at 24h in hu-C4BPxFH tg compared to wt BALB/c mice (Fig 6G, 6H and 6I).

## Discussion

GAS can bind both hu-C4BP and hu-FH like other pathogens, including *Neisseria meningitidis* and *Neisseria gonorrhoeae* [31, 32], *Moraxella catarrhalis* [33], *Candida albicans* [34] and *Haemophilus influenzae* [35, 36]. Based on studies *in vitro* that have shown down-regulation of C3 fragment deposition mediated by binding of FH and/or C4BP [17, 37, 38], it has been presumed that GAS may exploit these soluble inhibitors to escape complement attack *in vivo*, although direct evidence has been lacking. Here we present evidence that bacteria-bound complement inhibitors increase virulence and accelerate fatal infections *in vivo*. We have employed a novel mouse model that expresses hu-C4BP and/or hu-FH and have infected these animals with several GAS strains that differ in their ability to bind to these complement inhibitors. Our data provide evidence to support a general mechanism whereby recruitment of C4BP and FH to the GAS surface protects bacteria from clearance by phagocytes *in vivo* and contributes to increased morbidity and mortality in the infected experimental host.

Mice are not natural hosts for GAS infection, but can be experimentally infected with relatively high bacterial inocula [39]. We hypothesized that a GAS strain such as AP1, which binds human complement inhibitors via surface protein H, an M-like protein [14, 15], would show enhanced virulence in mice that expressed hu-C4BP and hu-FH. Indeed, the ‘double’ tg animals sustained higher bacterial burdens, displayed symptoms of bacterial sepsis and died more quickly than wt animals. Of note, the inoculum required to induce a lethal infection in the ‘double’ tg mice was reduced by more than 1 log_10_ compared to the inoculum required to kill wild-type animals. A second GAS strain (AP18) with similar hu-C4BP and hu-FH binding capacity as AP1 yielded similar survival results as AP1. In this case AP18 bind hu-C4BP and hu-FH directly via surface M protein [27, 29]. As a result, hu-C4BP and hu-FH binding GAS strains produced significantly more disease in hu-C4BPxFH tg animals than in wt mice. As ‘negative’ controls, we used GAS strains that were unable to bind hu-C4BP and hu-FH. We showed reduced mortality even at high inocula in the ‘double’ tg mice when compared to hu-C4BP and hu-FH-binding GAS strains in this model. Furthermore, we did not detect any differences in survival between wild type and hu-C4BPxFH tg animals that were challenged with strains unable to bind to these inhibitors (strain AP3 and the isogenic mutant derived from AP1, BM27.6). Taken together, these data strongly suggest that complement inhibitors exacerbate disease by binding GAS, but do not influence the course of GAS infection if the bacteria cannot recruit C4BP or FH to their surface.

Increased mortality of the double tg mice that were challenged with hu-C4BP/hu-FH-binding strains, AP1 and AP18, was not attributed to generalized defects in the immune systems caused by introduction of the human complement inhibitor transgenes for the following reasons. First, analysis of innate immune ligand-dependent cytokine release from peritoneal exudate cells (PECs) did not demonstrate differences between tg and wild type mice. Second, complement deposition on zymosan that resulted from incubation of zymosan with tg or wt mouse sera did not demonstrate differences between the sera. These findings suggest that our mouse model does not suffer from an apparent immune defect. Second, and as discussed above, the double tg mice did not suffer increased mortality compared to wt mice when challenged with strains that did not bind to hu-C4BP and hu-FH.

We postulate that exacerbation of infection in tg mice infected with GAS strains that bound complement inhibitors, resulted in impaired opsonization with mouse C3 fragments. We have shown previously that purified hu-C4BP injected in wt mice decreases complement activation via the classical pathway [22], which confirms that hu-C4BP regulates mouse complement. The β-chain of C4BP is not required for binding to GAS [26] and is not required for complement inhibition [40]; therefore the hu-C4BP molecule that lacks the β-chain—the form expressed by our tg animals, was fully functional as a complement inhibitor on the surface of GAS. [41]. Similarly, hu-FH bound to bacteria also inhibits non-human complement via the alternative pathway[42, 43].

Most pathogens activate complement via a combination of classical, lectin and alternative pathways (reviewed in [44]). Upon using hu-C4BPxFH double tg mice, we observed an additive effect of the two complement inhibitors, compared to using either hu-C4BP or hu-FH transgenic mice singly. Infection of singly transfected mice resulted in increased mortality in the respective mice but time to death was accelerated in the double tg mice.

Opsonization with C3 fragments is required for efficient uptake by phagocytes (reviewed in [20]). Thus, inhibiting complement activation impairs opsonization, results in diminished phagocytic uptake and decreases killing of pathogens. We showed that GAS strain AP1 recruited hu-C4BP and hu-FH to its surface, which reduced C3b/iC3b deposition on the bacterial surface and resulted in decreased phagocytosis of GAS both *in vitro* and *in vivo*. We saw diminished recruitment of neutrophils by GAS inoculated into the peritoneal cavity of ‘double’ tg mice and decreased uptake of bacteria by neutrophils that had been recruited. Diminished production of C3b results in decreased generation of both C5 convertase and C5a, a potent chemoattractant for neutrophils [45]. Impaired clearance of hu-C4BP and hu-FH-binding GAS was also reflected by greater CFU recovered from blood and other organs. Several of the cytokine levels that we measured were elevated in tg compared to wt mice, consistent with greater loads of organisms in tg mice [46].

Cytokines generated early, may be important in controlling bacterial dissemination but excessive and persistent production may be detrimental [47]. High levels of G-CSF in particular, generated within the first 24h have been reported to confer protection in mice infected with GAS [48] but in children, higher levels of pro-inflammatory cytokines generally, correlate with higher mortality from invasive GAS infections [49]. Infection of hu-C4BPxFH tg animals with strain AP1 resulted in elevation of most cytokine levels early at 2 hours, compared to wt animals; G-CSF levels at two hours were not different in C4BPxFH tg vs. wt mice but increased markedly in double tg animals at 24h. Cytokine levels, morbidity and fewer days to death, accompanied by increased bacterial burdens, were more pronounced in hu-C4BPxFH tg compared to wt mice. We hypothesize that failure to opsonize GAS and consequent reduced phagocytosis results in uncontrolled replication of GAS, which kills the host. A number of bacterial virulence factors are released, which lead to systemic toxicity, coagulopathy, hypotension, septicemia, tissue damage and finally multi organ failure [11, 50, 51].

Our data differ from a previously published study that did not demonstrate accelerated mortality during acute GAS infection in C57BL/6, mouse-FH KO, transgenic (tg) mice that expressed only chimeric human/mouse FH (SCRs 6–8 were derived from human FH) [28]. Mortality was not affected despite evidence of binding of hu-SCR 6–8 to the M protein (M5) of the infecting strain [28]. This study used a C57BL/6 tg mouse model whose levels (200–210 μg/ml) of chimeric FH had been reported earlier [52] to be similar to FH levels in wt C57BL/6 mice. These FH levels were lower than those in our tg mice; 379.9 μg/ml in FH tg mice and 291.5 μg /ml in ‘double’ tg C4BPxFH. These levels were similar to levels reported in human (320 ± 71.4 μg/ml in plasma taken from 358 individuals [53]). The higher levels may have been important to display the completely virulent phenotype in mice. Furthermore, chimeric FH, expressing hu-SCRs 6–8 [28], may also have undergone unique conformational changes, distinct from those that occur with native hu-FH [54], which may be important in maintaining physiologic function. Differences in mouse strains (C57BL/6 mice were used in the chimeric FH study [28]; we used BALB/c mice), bacterial strains and routes of inoculation all could have contributed to differences in our results compared to those of the previous study [28].

In conclusion, we have demonstrated a detrimental influence of human complement inhibitors FH and C4BP in overcoming experimental GAS sepsis *in vivo*. Our data suggest a pivotal role for complement inhibitors on GAS strains that bind these inhibitors to their surface. Our novel hu-C4BPxFH tg animal infection model may prove invaluable in studies of GAS pathogenesis and in the development of vaccines and therapeutics that incorporate a ‘human’ context.

## Materials and Methods

### Antibodies

The following antibodies were used for ELISA measurements: 10 μg/ml rabbit anti hu-C4BP PK9008, (homemade, capture Ab); 0.5 μg/ml mouse anti hu-C4BP MK104, (homemade, detection Ab); 10 μg/ml mouse anti hu-fH MRC OX24, (homemade [55], capture Ab); 5 μg/ml sheep anti human-Factor H (Abcam, ab8842; detection Ab). C4BP and FH detection antibodies were secondarily detected using anti sheep IgG-HRP or anti mouse IgG-HRP (DAKO, P0163 and P0260).

For flow cytometry analysis, the following antibodies were used: mouse anti human-C4BP MK104 either unconjugated or conjugated to biotin; mouse anti human-Factor H MRC OX24 unconjugated or conjugated to biotin; rabbit anti mouse-C4BP (homemade) conjugated to Dylight 647; mouse monoclonal anti mouse-Factor H (Hycult, HM1119) conjugated to biotin; goat anti mouse-C3c (Nordic Immunology, GAM/C3c/7S); anti mouse C3 FITC (MP Biomedicals #0855500) anti mouse Ly-6G brilliant violet 421 (BioLegend, #127627); anti mouse Ly-6C PerCP/Cy5.5 (BioLegend, #128011); anti mouse CD11c (BioLegend, #117317); anti mouse I-A/I-E brilliant violet 510 (BioLegend, #107635); anti mouse CD64 APC (BioLegend, #139305); anti mouse/human CD11b APC/Cy7 (BioLegend, #101225). Unlabeled primary antibodies used for detection of the nominal targets in FACS were themselves bound and detected using donkey F(ab’)_2_-anti mouse IgG-PE (Thermo, #31860) or donkey F(ab’)_2_-anti goat-IgG-PE (eBioscience, #12-4012-87). Final reactions that measured biotin labeled antibody binding were disclosed with streptavidin-Dylight 650 (Pierce, #84547) or streptavidin-PE (eBioscience, #12-4317-87).

For western blot analysis of human C4BP in mouse serum we used mouse anti hu-C4BP MK104 coupled to biotin detected by Dylight 649 Streptavidin (BioLegend, 405224). Hu-FH in mouse serum was detected using goat anti human FH (Calbiochem, #341276) and Alexa Fluor 647 donkey anti goat IgG (Life Technologies, A21447). Western blots were read using Typhoon FLA 9500 (GE Healthcare).

### Bacterial strains

*Streptococcus pyogenes* AP1 (strain 40/58, serotype M1), AP3 (strain 4/55, serotype M3) and AP18 (strain 8/69, serotype M18) were obtained from the WHO Collaborating Centre for Reference and Research on Streptococci, Prague, Czech Republic. BM27.6 is an isogenic mutant of AP1 lacking protein H [56]. Binding of human soluble complement inhibitors, C4BP and FH, to each strain is summarized in Table 1. Streptococcal strains were grown in Todd-Hewitt broth (THB) and *Moraxella catarrhalis* RH4 (control strain) in brain-heart infusion (BHI) broth overnight at 37°C and 5% CO_2_ without shaking. Cultures were then diluted to OD_600_ = 0.1 in corresponding fresh medium and incubated again at 37°C and 5% CO_2_ without shaking, until exponential growth at OD_600_ = 0.3–0.4 was achieved. Bacteria were harvested and washed with 1× PBS prior to use.

### CovRS sequencing in GAS strains

Genomic DNA from GAS AP1, AP3 and AP18 strains was isolated using a DNeasy blood and tissue kit (Qiagen) according to manufacturers instructions. The covRS operon was amplified (for primers used see S1 Table) by PCR and subsequently subjected to Sanger sequencing.

### Production of hu-C4BP, hu-FH and hu-C4BPxfH transgenic mice

All animals were housed and bred under SPF conditions in the animal facility at the University of Massachusetts Medical School Worcester (UMMS), USA.

Production of hu-FH transgenic mice has been described previously [21]. To generate human C4BP transgenic mice, full-length cDNA encoding human C4BP (1.8 kbp) was subcloned into the *Eco*RI site of the expression vector pCAGGS [57]. A CMV enhancer and chicken β-actin promoter sequences are located upstream of the *Eco*RI site in pCAGGS and a rabbit β-globin polyA sequence is located downstream of the *Eco*RI site. The resultant plasmid, pCAGGS-human C4BP, was digested with *Sal*I and *Hin*dIII to isolate the transgenic cassette fragment that consisted of the CMV enhancer, the chicken β-actin promotor, the human C4BP cDNA and the rabbit β-globin poly(A) sequence. The isolated 4 kb *Sal*I and *Hin*dIII fragment was purified and microinjected into mouse embryos from BALB/c mice. Mouse embryos were implanted into pseudo-pregnant female BALB/c mice (Charles River Breeding Laboratories) at the UMMS Transgenic Facility. Human C4BP transgenic mice initially were identified by PCR analysis using genomic DNA prepared from mouse-tails. A region inside human C4BP was amplified by PCR using primers C4BP-*EcoRI* and C4BP-*NotI* to yield a 383-bp product (Fig 1B; for primer sequence see S1 Table). Amplified products were resolved by electrophoresis on 2% TAE agarose gels and visualized with ethidium bromide staining under UV light. Expression of human C4BP in sera of pups was detected by Western blotting using affinity purified rabbit anti-human C4BP. FH and C4BP transgenic mice were bred together to create double transgenic mice. To assess serum levels of hu-C4BP and hu-FH sandwich ELISAs (see antibodies) were performed.

### Serum preparation

Animals were anesthetized with Isoflurane and blood was drawn by cardiac heart puncture. Blood samples were kept on ice for 30 min and allowed to clot before centrifuging for 10min at 1700 x g, 4°C. Serum was separated, aliquoted and directly frozen at -80°C until use.

### Generation of bone marrow derived macrophages

Bone marrow was extracted from femurs and tibias of 3 euthanized mice and plated onto DMEM/high glucose supplemented with 10% FCS, 5% horse serum and 2500U/ml M-CSF. Bone marrow was incubated for 7 days at 37°C, 5% CO_2_ to allow differentiation into bone marrow derived macrophages (BMDM). Cells were washed 3x with ice cold PBS, pooled and frozen in DMEM/high glucose supplemented with 10% FCS, 5% horse serum, 2500U/ml M-CSF and 10%DMSO until further use. Flow cytometry analysis showed a uniform population of CD11b^high^, F4/80^high^ MHC^low^ cells, indicating macrophages.

### Complement deposition assays

Harvested bacteria were incubated with increasing amounts of either normal human serum or mouse serum for 1 h at 37°C, 5% CO_2_. Bacteria were washed three times with 1× PBS before and after each staining step. Bacteria were stained as indicated for either human or mouse C4BP, FH and C3b. Unconjugated primary antibodies were detected either with secondary antibodies or streptavidin coupled to PE or Dylight 649 (Pierce). The amount of surface bound complement was measured using a Cyflow space flow cytometer (Partec).

Complement deposition on zymosan particles was performed as described previously [58]. Briefly, zymosan was incubated in either wt or hu-C4BPxFH tg mouse sera (final serum concentration 20%) for 30 min at 37°C. Controls included zymosan incubated with wt mouse serum containing 10 mM EDTA to block activation of all pathways of complement. After washing, particles were stained for deposited C3 and analyzed using an LSRII flow cytometer (BD).

### *In vitro* infection experiments

#### *In vitro* phagocytosis

Frozen stocks of BMDMs were defrosted and cultured over night in DMEM + 10% FCS at 37°C one day prior to use. On the day of the experiment, 10^4^ BMDMs were seeded into 96-wells and allowed to attach prior to infection. GAS AP1 (for preparation see Complement deposition assays) was used in DMEM + 10% FCS at an MOI of 10:1. Additionally, 10% of the serum to be tested was added to the respective well. BMDMs were allowed to phagocytose GAS AP1 for 60 min. at 37°C 5% CO_2_, then 300μg/ml gentamicin was added for 60 min to kill extracellular bacteria. After three washes with 1x PBS, BMDMs were lysed with 0.1% Triton X-100 in H_2_O. Bacteria were serially diluted, plated onto blood agar plates, incubated at 37°C 5% CO_2_ for 18h and enumerated.

#### Cytokine release

Mice were injected with 4% thioglycollate and peritoneal exudate cells (PECs) were harvested 4 days later by lavage. Exudate cells (>90% macrophages) were plated at 5x10^5^ cells per well in 24-well tissue culture plates in DMEM containing 10% FCS. PECs were challenged with LPS (10 ng/ml), Pam_2_CSK_4_ (100 ng/ml), poly dAdT (1 μg/ml with lipofectamine), Sendai virus (40HA) or GAS AP1 or *Neisseria gonorrhoeae* (*Ng*) bacteria (MOI = 1 cfu/cell). Culture supernatants were collected 24h later to analyze for released IL-6 and RANTES by ELISA. For bacterial stimulation, the bacteria were added for 2h, PECs were washed and fresh DMEM containing 10% FCS and penicillin/streptomycin was added. Lipopolysaccharide (LPS) from *Escherichia coli* O111:B4 was obtained from Sigma (St. Louis, MO) and phenol extraction was performed prior to use as previously described [59]. Pam_2_CSK_4_ was obtained from EMC Microcollections (Tuebingen, Germany). IL-6 and MCP-1 ELISA kits were obtained from BD Pharmingen.

### *In vivo* infection experiments

#### *In vivo* phagocytosis

Animals were infected intraperitoneally with 100μl bacterial suspension in PBS containing 5x10^7^ CFUs of CFSE (Sigma, #21888) labeled *S*. *pyogenes* AP1 or BM27.6. After 2h, animals were euthanized and cells were harvested by flushing the peritoneum with ice cold PBS + 2mM EDTA + 2% FCS. Recovered cells were washed once in PBS + 2mM EDTA +2% FCS and stained with antibody for FACS analysis. Prior to staining, non-specific binding of antibodies was blocked using truStain FcX (anti mouse CD16/32; BioLegend, #101319). Cells were analyzed using an LSRII flow cytometer (BD).

#### Survival analysis

Animals were infected intravenously via lateral tail vein injection with 100μl bacterial suspension in PBS containing the indicated CFUs of different *S*. *pyogenes* strains. Infected animals were closely monitored for signs of disease (vocalization, socialization, posture, pili erection and respiration) and maintained for up to eight days; gravely moribund mice were sacrificed.

#### Bacterial dissemination

In a separate experiment bacterial burdens were enumerated in different organs at 2h and 24h from mice that had been inoculated with 5x10^6^ CFUs of GAS AP1. At each time point, half the infected wild type and transgenic animals were euthanized according to IACUC guidelines. Spleens, livers, kidneys and blood were harvested, complete organs weighed and homogenized in 2 ml ice cold PBS; serial dilutions were plated on blood agar plates (TSA II 5% SB, BD), incubated in 5% CO_2_ at 37°C over night and numbers of colonies enumerated.

#### Cytokine analysis

Sera were also collected from animals at 2 and 24 hours, the times when animals were euthanized and sera stored at -80°C. Serum cytokine levels were determined using a cytokine multiplex assay (Bio-Rad) according to manufacturers instructions.

### Statistical analyses

Statistical analysis was performed using GraphPad Prism 5.0f software. Samples were tested for normal distribution using a D’Agostino and Pearson omnibus normality test. According to the result, samples were then analyzed either using a parametric or non- parametric test as indicated in corresponding figure legends.

### Ethical statement

The Institutional Animal Care and Use Committee in Worcester, MA, USA, approved all animal experiments.

Human serum was prepared from venous blood of healthy volunteers according to the recommendations of the local ethical Committee in Lund, Sweden. Written informed consent was obtained; all investigations were conducted according to the principles of the Declaration of Helsinki.

#### Accession numbers NCBI

human C4BP Accession: AAA36507.1 (alpha chain) + NP_001017367.1 (beta chain)

human Factor H Accession: CAA68704.1

human C3 Accession: AAA85332.1

mouse C4BP Accession: NP_031602.3

mouse Factor H Accession:AAH66092.1

mouse C3 Accession: AAH43338.1

protein H Accession: CAA01972.1

## Supporting Information

S1 Fig BALB/c and C4BPxFH tg mice show similar response to TLR stimulation. (**A**) RANTES and (**B**) IL6 levels produced by thioglycollate-elicited peritoneal macrophages derived from BALB/c and hu-C4BPxFH tg mice stimulated with different ligands. The ligands tested were LPS (TLR4 ligand), Pam2CSK4 (TLR2 ligand), lipofectamine carrier, lipofectamine + dAdT (STING ligand), Sendai virus (RIG-I ligand), and whole bacteria GAS (AP1) and gram-negative bacteria (*Neisseria gonorrhoeae*, N.G.). 2 individual mice per group are shown. Statistical analysis: 2-way ANOVA with Bonferroni’s post-test
(EPS)

S2 Fig Gating strategy to identify neutrophils elicted in the peritoneum by IP infection. (**A**) Peritoneal cells elicited by infection were gated to exclude cell debris, cell doublets and larger aggregates and dead cells. Neutrophils were defined as cells that stained CD64^intermediate^ and Ly-6G^high^. Gating of a representative experiment is shown.
(EPS)

S3 Fig GAS AP1 but not BM27.6 reduce neutrophil recruitment and evade phagocytosis in hu-C4BPxFH tg mice. BALB/c and hu-C4BPxFH tg mice were injected i.p. with CFSE-labeled GAS AP1 (**A** and **C**; 5 BALB/c and 8 hu-C4BPxFH tg mice) or GAS BM27.6 (**B** and **D**; 6 BALB/c and 5 hu-C4BPxFH tg mice). (**A**) and (**B**) show the proportion of neutrophils that infiltrated the peritoneal cavity. (**C**) and (**D**) show the percent of neutrophils that phagocytosed GAS. All results are expressed as the Mean ± SEM. Statistical analysis: Student’s t-test.
(EPS)

S4 Fig Hu-C4BP and hu-FH binding to different GAS strains. GAS strains AP1, BM27.6, AP3 and AP18 were incubated in increasing amounts of hu-C4BPxFH tg serum prior to FACS analysis to detect surface bound: (**A**) hu-C4BP or (**B**) hu-FH. Results are expressed as the Mean ± SD of three independently performed experiments.
(EPS)

S5 Fig GAS strains unable to bind hu-C4BP and FH show similar rates of mortality in BALB/c and hu-C4BPxFH tg mice. Wild-type BALB/c (n = 10 in each group, unless stated otherwise) mice were infected i.v. with either: (**A**) 2x10^7^ CFU AP3 (7 BALB/c and 7 hu-C4BPxFH tg); (**B**) 1x10^8^ CFU AP3; (**C**) 1x10^7^ CFU BM27.6 (22 BALB/c and 12 hu-C4BPxFH tg) or (**D**) 5x10^8^ CFU BM27.6. In each experiment, both groups of animals showed similar signs of morbidity and mortality. Statistical analysis in all graphs was performed using the Mantel-Cox test.
(EPS)

S1 Table Primers used for genotyping of transgenic mice and bacterial strains. (DOCX)
