# Supplementary material for: Virulence of Group A Streptococci Is Enhanced by Human Complement Inhibitors
Source: PLoS Pathog. 2015 Jul 22;11(7):e1005043. doi: 10.1371/journal.ppat.1005043 (PMC4511809; doi:10.1371/journal.ppat.1005043)
Supplement: S1 Table — (DOCX) [file ppat.1005043.s006.docx]

S1 table

| Name | Target | Sequence |
| --- | --- | --- |
|  |  |  |
| C4BP EcoRI | C4BP | 5'- GAA TTC CAA TTG TGG TCC TCC ACC CAC TT-3' |
| C4BP NotI | C4BP | 5'- GCG GCC GCT TCA CAT TGT GGG AGA GGA TGA CT -3' |
| SCR7F | FH | 5’- CAT CCT GGC TAC GCT CTT CCA AAA-3’ |
| SCR8R | FH | 5’-ATC TAA TTG ATC CTG ATG TTT CAC C- 3’ |
| CovRS_for | CovRS | 5’- CCA TAG AGG GCA GAG AAG G-3’ |
| CovRS_mid | CovRS | 5’- ATC TCC GCG GCA AAA TTG AC -3’ |
| CovRS_rev | CovRS | 5’- CAT CAG CTT CTA ACC AGT TGT G-3’ |

S1 table: Primers used for genotyping of transgenic mice and bacterial strains.
